# Delirium in the elderly: A systematic review of pharmacological and non-pharmacological treatments

**DOI:** 10.1590/1980-57642016dn11-030009

**Published:** 2017

**Authors:** Cecília Carboni Tardelli Cerveira, Cláudia Cristina Pupo, Sigrid De Sousa dos Santos, José Eduardo Mourão Santos

**Affiliations:** 1 MD, Unidade Básica de Assistência à Saúde, Universidade de São Paulo – Campus de São Carlos, SP, Brazil.; 2 MD, Departamento de Medicina, Centro de Ciências Biológicas e da Saúde, Universidade Federal de São Carlos, SP, Brazil.; 3 MD, PhD, Departamento de Medicina, Centro de Ciências Biológicas e da Saúde, Universidade Federal de São Carlos, SP, Brazil.

**Keywords:** delirium, elderly, pharmacological, non-pharmacological, systematic review, delirium, idoso, farmacológico, não-farmacológico, revisão sistemática

## Abstract

Delirium is a common disorder associated with poor prognosis, especially in the elderly. The impact of different treatment approaches for delirium on morbimortality and long-term welfare is not completely understood.

**OBJECTIVE:**

To determine the efficacy of pharmacological and non-pharmacological treatments in elderly patients with delirium.

**METHODS:**

This systematic review compared pharmacological and non-pharmacological treatments in patients over 60 years old with delirium. Databases used were: MEDLINE (PubMed), EMBASE, Cochrane CENTRAL and LILACS from inception to January 6^th^, 2016.

**RESULTS:**

A total of ten articles were selected. The six non-pharmacological intervention studies showed no impact on duration of delirium, mortality or institutionalization, but a decrease in severity of delirium and improvement in medium-term cognitive function were observed. The most commonly used interventions were temporal-spatial orientation, orientation to self and others, early mobilization and sleep hygiene. The four studies with pharmacological interventions found that rivastigmine reduced the duration of delirium, improved cognitive function and reduced caregiver burden; olanzapine and haloperidol decreased the severity of delirium; droperidol reduced length of hospitalization and improved delirium remission rate.

**CONCLUSION:**

Although the pharmacological approach has been used in the treatment of delirium among elderly, there have been few studies assessing its efficacy, involving a small number of patients. However, the improvements in delirium duration and severity suggest these drugs are effective in treating the condition. Once delirium has developed, non-pharmacological treatment seems less effective in controlling symptoms, and there is a lack of studies describing different non-pharmacological interventions.

## RESUMO

*Delirium* é uma condição comum associada com prognóstico pobre, especialmente em idosos. O impacto das diferentes abordagens de tratamento na morbi-mortalidade e bem-estar de longo prazo não é completamente compreendido.

**OBJETIVO:**

Determinar a eficácia dos tratamentos farmacológico e não-farmacológico em pacientes idosos com *delirium*.

**MÉTODOS:**

Esta revisão sistemática comparou tratamentos farmacológicos e não-farmacológicos em pacientes com idade superior a 60 anos com *delirium.* As bases de dados usadas foram: MEDLINE (PubMed), EMBASE, Cochrane Central e LILACS do início até 6 de janeiro de 2016.

**RESULTADOS:**

Dez artigos foram selecionados. Seis estudos com intervenções não farmacológicas não mostraram impacto na duração, mortalidade ou institucionalização, mas houve redução na gravidade do *delirium* e memlhora na função cognitive de médio prazo. As intervenções mais comumente utilizadas foram orientação temporo-espacial, orientação para si e outros, mobilização precoce e higiene do sono. Os quarto estudos com intervenções farmacológicos acharam que a rivastigmina reduziu a duração, melhorou a função cognitive e reduziu a sobrecarga do cuidador, olanzapine e haloperidol diminuíram a gravidade de *delirium* e o droperidol diminuiu a duração da hospitalização e na taxa de remissão.

**CONCLUSÃO:**

Embora a abordagem farmacológica tem sido usada no tratamento de *delirium* em idosos, há estudos que avaliam sua eficácia com limitado número de pacientes. Todavia, a melhora na duração e gravidade sugerem que estas drogas são efetivas no tratamento desta condição. Uma vez que o *delirium* esteja instalado, o tratamento não farmacológico parece ser menos efetivo no controle dos sintomas, há uma falta de estudos que discriminem as diferentes intervenções não-farmacológicas.

## INTRODUCTION

Delirium is an acute confusional state characterized by consciousness disorder, decline in cognitive function and attention, abrupt onset and fluctuating course.^1^ The condition is associated with a rapid reduction in brain function and is usually triggered by diseases with systemic involvement. It has a considerable impact on morbidity and lethality, especially among the elderly.^1^^-^ 4

The incidence of delirium is 1-2% in the general population, 22% in hospitalized patients, 11-62% in postoperative patients (depending on type of surgery and study population) and up to 80% among patients in intensive care units.^2^^,^ 5 ^-^ 7 In the elderly, the incidence reaches 50% in hospitalized patients.^6^^,^ 7 Nevertheless, many cases go undiagnosed.

Delirium is associated with many negative outcomes including increased length of hospital stay and risk of complications; higher mortality (8% versus 1%), institutionalization rates (16% versus 3%),^7^ and risk of permanent cognitive decline.^4^^-^ 10 In intensive care units, the condition is associated with an increased length of stay and longer duration of mechanical ventilation.^11^

The presence of delirium accelerates the ageing of the brain, elevates the risk of dementia in predisposed individuals and can mask prior cognitive impairment not yet diagnosed.^8^^,^ 9 ^,^ 12

The major risk factors for delirium are previous cognitive impairment, history of alcohol abuse and advanced age (>70 years).^6^^,^ 13 The presence of dementia is the major risk factor of delirium in the elderly. Polypharmacy (use of five or more medications) is the commonest independent and reversible risk factor of delirium. Sedatives, analgesics and anticholinergic drugs are the most commonly involved. Other risk factors involved are: severity of the underlying disease, infections, fractures at admission and physical restriction. The duration of delirium in the elderly is generally longer and the symptoms more severe.^2^^,^ 6

The non-pharmacological approach has been the most used strategy for preventing delirium.^6^ A non-pharmacological intervention package known as the Hospital Elder Life Program – HELP (cognitive impairment management, sleep hygiene, early mobility, visual and hearing support, hydration) has demonstrated effects on the incidence and total days with delirium. However, once delirium has developed, HELP seems to have no impact on severity and recurrence.^13^^,^ 14

Studies using different pharmacological approaches for delirium prevention (haloperidol, olanzapine, risperidone, rivastigmine, ketamine, dexmedetomidine, morphine, donepezil and midazolam) have not found sufficient evidence of effectiveness.^6^ However, melatonin and its agonist ramelteon have been found to be potentially beneficial in the prevention of delirium in the elderly.^5^

Treatment of delirium is mainly based on resolution of the underlying condition combined with non-pharmacological interventions and specific pharmaceutical interventions.^15^ Consequently, delirium diagnosis and early identification of its causal and predisposing factors depend on healthcare team training. After discharge, patients who have developed delirium require monitoring given the high possibility of a further episode.^2^ To date, no specific treatment for delirium has been approved by the Food and Drug Administration.^16^^,^ 17

For patients in psychic distress or too restless, at risk of harming themselves, caregivers or health professionals, antipsychotics have been recommended based on clinical evidence and expert opinion.^2^ Haloperidol, a typical antipsychotic and neuroleptic, has been the most studied and routinely used medication. It blocks cortical and nigrostriatal dopamine receptors (D2 antagonist), and disinhibits acetylcholine. It should be initiated at the smallest possible dose for the shortest possible period. Side effects include extrapyramidal symptoms, akathisia, neuroleptic malignant syndrome, tardive dyskinesia, glucose and cholesterol changes, cardiac arrhythmias, and venous thromboembolism.^17^

In a systematic review of studies comparing atypical antipsychotics (amisulpride, quetiapine, olanzapine and risperidone) with typical antipsychotics (haloperidol) for the treatment of delirium in a wide range of clinical conditions, all medications have been proven effective and safe with no significant difference between agents.^1^

Perioperative melatonin has been compared to no drug (control), midazolam or clonidine in prevention and treatment of postoperative delirium. Patients receiving melatonin had a lower incidence of postoperative delirium. In addition, postoperative delirium was successfully treated with melatonin in 58% of patients. However, this response was not controlled, and it is difficult to impute causality.^18^^,^ 19

There is no evidence to support the use of benzodiazepines for the treatment of cases of delirium not related to alcohol withdrawal. These agents may have a deleterious effect, triggering delirium, increasing the risk of falls, as well as causing changes in memory and withdrawal symptoms.^2^^,^ 15

Non-pharmacological interventions, characterized by a multi-professional activity, have been the main approach used in patients with delirium. Although there are many therapeutic approaches for delirium (cognitive impairment management, sleep hygiene, early mobility, visual and hearing support, hydration, environment interventions, orientation interventions, familiarity interventions, communication strategies, pain control etc.); the importance, impact, and specificity of each of these interventions on morbidity, mortality and quality of life after discharge is unclear.^15^^-^ 17

The main goal of the present systematic review was to identify studies investigating the effectiveness of pharmacological and non-pharmacological interventions on the duration of delirium in patients aged 60 years or older. As secondary goals, the effects of these interventions on length of hospital stay, lethality, incidence of complications and functional decline were evaluated.

## METHODS

Our study was designed according to the recommendations of Higgins & Green (2011) "Cochrane handbook for systematic reviews of interventions".^20^ The databases used were MEDLINE (PubMed), EMBASE, Cochrane Library (CENTRAL) and LILACS. All databases were searched from inception to January 6^th^, 2016. There was no restriction on language. The following major Medical Subject Headings terms were included: ("delirium" OR "acute confusion" OR "confusion state" OR "confusional state" OR "acute confusion state" OR "acute confusional state") AND ("elderly" OR "aged" OR "ancient" OR "geriatric") AND ("treatment" OR "therapy"). The filters "Clinical Trial" OR "Trial" were used for MEDLINE; "Trial" for the Cochrane Library; and "controlled clinical trial" OR "randomized controlled trial" for EMBASE. No filter was used for LILACS.

Eligible studies for inclusion were randomized or controlled clinical trials in which patients were 60 years or older with the diagnosis of delirium based on the *Diagnostic and Statistical Manual of Mental Disorders* (DSM), *International Classification of Diseases-10* (ICD-10) or the use of a validated instrument for the diagnosis of delirium.

The primary outcome was the duration of delirium. The secondary outcomes were: length of hospital stay, need of physical restraint, lethality, functional decline and the incidence of complications.

The decision to exclude studies was reached through consensus and based on the established criteria, including participants, interventions, comparisons, outcomes, and study design (PICOS). A third reviewer (SSS) was consulted in case of doubt or disagreement between the reviewers.

Acceptable studies had a clear description of design, age range, diagnosis, intervention and outcome. The review process excluded studies with unclear designs, improper control group, younger or adult-only samples, insufficient outcome data, unclear diagnosis of delirium at study admission, or insufficient outcome data.

As required, non-English language articles were translated into English using an online translation service (Google Translator), with subsequent revision by a native speaker (Russian, Chinese and Korean).

The data from the selected articles were extracted using a standard form, including identification, study design, characteristics of the study population, type of intervention, outcomes, main results and comments. Study authors were contacted for additional data when needed.

## RESULTS

**Figure 1:**
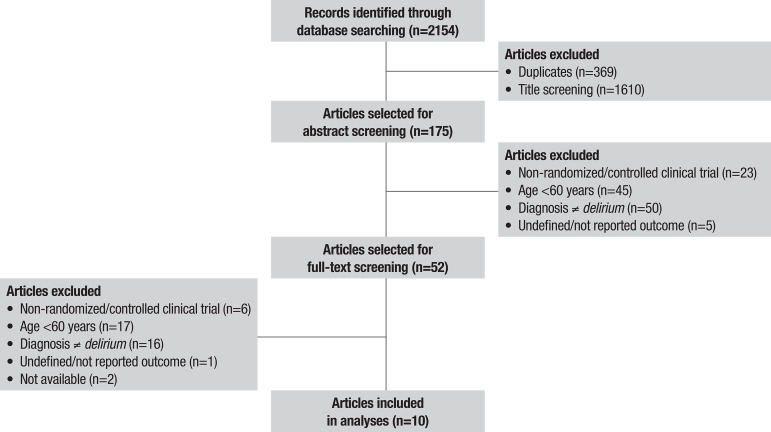
PRISMA flow diagram of study inclusion and exclusion.

A total of 2154 articles were selected for review, comprising 431 studies in PubMed; 1170 studies in EMBASE; 481 studies in the Cochrane Library (CENTRAL); and 72 studies in LILACS. We selected 10 studies evaluating 1588 patients. The selection algorithm is depicted in Figure 1.

The selected studies are described in Table 1, according to study design, clinical and epidemiological characteristics of the study population, prior prevalence of dementia, delirium diagnostic criteria, interventions, observed outcomes and results.

**Table 1:** Summary of methods and results of studies included in the systematic review.

Author, Year	Type (P, NP)	Study population, n, mean age, male	Intervention/control	Results	Limitations
Chong et al., 2014^21^	• NP	• 320 geriatric patients (IW 234/ CW 39/ HCW 47), 84 years, 39%	• I: HELP program, bright light therapy (2,000-3,000 lux 6-10 pm)	• No impact on *delirium* duration, length of stay, nosocomial infection, mortality or discharge destination • Improvement in functional status (MBI 19.2 vs 15.2 vs 7.5, p<0.05), restraint rate (0 vs 23.1 vs 44.7%, p<0.05), and pressure ulcer (4.1 vs 1.3 vs 9.1% p<0.05)	• Small control group; dementia IW>CW. • Statistical differences among the three groups (IW, CW and HCW, respectively)
Cole et al., 1994^22^	• NP	• 88 clinical patients (IW 42/ CW 46), 86.1 years, 35%	• I: psychiatric and geriatric specialist, EI, OI, FI,CI, early mobility	• No impact on restraint rate, length of stay, mortality, discharge dependence, and cognitive decline in 8 weeks. • Improvement in cognitive decline in 2 weeks (SPMSQ, p<0.05)	• Small sample, very ill patients, high mortality rate (35%) sub diagnosis of *delirium*
Cole et al., 2002^23^	• NP	• 227 clinical patients (IW 113/ CW 114), 82 years, 46%	• I: psychiatric and geriatric specialist, EI, OI, FI, CI, early mobility	• No impact on cognitive decline in 8 weeks, *delirium* severity, functional status, length of stay, discharge rate, and mortality	• Same staff care between IW and CW
Hu et al., 2006^24^	• P	• 175 university hospital patients (IP1 72/IP2 74/ CP 29), 73.8 years, 63%	• I1: haloperidol 2.5-10mg IM per day; I2: olanzapine 1.25-20 mg per day PO or SL; C: no drug for CNS	• Improvement in severity of mental illness in 7 days (I1>I2>C, p<0.01), global recovery of mental disease in 7 days (I1>C,p<0.01), DRS in 1 day (I2<I1<C,p<0.01), DRS in 7 days (I1<C,p<0.01)	• Non intention-to-treat protocol
Litvinenko et al., 2010^25^	• P	• 68 ischemic stroke patients (IP 21/ CP 47), IG, IG	• I: rivastigmine 9-12mg PO per day for 14-25 days followed by patch of 9.5 mg per day for 8 months; C: haloperidol as needed	• Improvement in *delirium* duration 3 -12 days vs 5 – 28 days, p<0.001), FAB (14,8 vs 12, p< 0.001), MMSE (26.7 vs 22.5 p<0.001), 10-word memorizing test (3.5 vs 2.4, p<0.05), and caregiver burden in 3 and 6 months (p<0.05)	• Open label study, lethality I=22.8%, C=36.2% not compared; dementia prevalence ignored
Marcantonio et al., 2010^26^	• NP	• 457 clinical or surgical patients (IW 282/ CW 175), 84 years, 35%	• I: systematic assessment of *delirium* causes; OI; EI; CI; PC; UF; caregiver guide	• No impact on *delirium* persistence or mortality	• Incentive payment for *delirium* diagnosis in IW
Mudge et al., 2013^27^	• NP	• 46 clinical patients (IW 19/ CW 27), 83.1 years, IG	• I: staff education and training; judicious use of drugs for CNS; hydration; EI; OI; CI;FI; PC; UF; caregiver guide; catheter control; staff and caregiver guide	• No impact on mortality and falls. IW more likely to receive psychogeriatric consultation (32% vs 11%, p = 0.04), and with a longer length of acute stay (median IQR: 16 vs 8 days, p<0.01)	• Daily evaluation of *delirium* was not done, implementation of interdisciplinary team care in both wards
Niu et al., 2014^28^	• P	• 18 postoperative patients (IP 9/ CP 9), 79.5 years, IG	• I: droperidol 5mg IM; C: no drug for CNS	• Improvement in length of hospital stay (p<18.3 vs 21.1 days, p<0.05); *delirium* remission (6 vs 1 patient, p<0.05)	• Small sample; dementia prevalence ignored
Overshott et al., 2010^29^	• P	• 15 clinical patients (IP 8/ CP 7), 83 years, 53.3%	• I: rivastigmine 1.5-3.0 PO per day; C: placebo	• Improvement in *delirium* remission rate in 28 days (8 vs 3 patients, p=0.03) • No impact on *delirium* duration	• Small sample; low rivastigmine dose; CAM obtained from ward nurse
Pitkälä et al., 2006^30^	• P/NP	• 174 clinical and surgical patients (IP 87/ CP 87), 83.6 years, 26%	• I: preference for atypical antipsychotics as needed; OI; FI; physiotherapy; calcium and vitamin D supplements; hip protectors; nutritional supplements; cholinesterase inhibitors as needed; geriatric specialist; C: conventional neuroleptics as needed	• Decrease in time to *delirium* recovery (sustained improvement of at least 4 points on MDAS) (p<0.002); improvement on MMSE in 6 months (18.4 vs 15.8, p=0.047). Higher number of days in acute care (52 vs 42, p=0.032) • No impact on functional status or combined endpoint (permanent institutional care or death in 3 and 6 months)	• Very frail patients; implementation of interdisciplinary team care in both groups

P: pharmacological; NP: non-pharmacological; IW: intervention ward; CW: control ward; HCW: historical control ward; IP: intervention patient; CP: control patient; IG: ignored; I: intervention; C: control; HELP program (cognitive impairment management, sleep hygiene, early mobility, visual and hearing support, hydration); EI: environment intervention (light, silence, radio, television); OI: orientation intervention (clock, calendar, day's schedule chart, visual and hearing support, language interpreters); FI: familiarity intervention (objects and family members); CI: communication intervention (visual contact, empathy, calm speech); PC: pain control; UF: urinary and fecal function; IM: intramuscular; PO: orally; SL: sublingually; CNS: central nervous system; DD: delirium duration (days); DRS: delirium rating scale; FAB: frontal assessment battery; MBI: Modified Barthel Index; SPMSQ: Short Portable Mental Status Questionnaire); MMSE: mini-mental state examination; MDAS: Memorial *Delirium* Assessment Scale; IQR: interquartile range; vs: versus.

A meta-analysis could not be performed due to several factors, including the variability in interventions proposed as non-pharmacological treatments, in doses and types of drugs used as pharmacological treatment, intervention strategies (individual or specific ward), parameters and time interval used for outcome evaluation, and type of comparison group employed.

## DISCUSSION

Both pharmacological and non-pharmacological interventions have shown impact on health status of elderly patients with delirium. Non-pharmacological interventions, while not significantly affecting the duration and severity of delirium, or mortality; did contribute to functional improvement in the medium term and reduced complications. Several medications were studied to control agitation of hyperactive delirium, with demonstrable improvement in delirium severity, duration and remission rate, positively affecting length of hospital stay and caregiver burden.

Interventions that combine cognitive impairment management, sleep hygiene, early mobility, visual and hearing support, and hydration care (HELP protocol) with light therapy have contributed to significant improvement in functional status and to absence of physical restraints for the care of delirium in geriatric wards.^21^

Strategy combining orientation interventions (clock, calendar, day's schedule chart, visual and hearing support, language interpreters); familiarity interventions with objects and family members; physiotherapy; hip protectors, and nutritional supplements with the prescription of cholinesterase inhibitors as needed and restriction of the use of neuroleptics provided more pronounced and rapid reduction in the severity of delirium and cognitive improvement after discharge.^30^

Intervention combining entertainment (radio, television); sleep hygiene (light and sound); orientation interventions; familiarity interventions with the presence of objects and people they know; attention to visual and auditory acuity, communication intervention with visual contact, empathy, calm speech; early mobility stimulation, and psychiatric and geriatric specialist consultation was associated with more rapid cognitive improvement.^29^

The reviewed studies have a small number of participants, a possible underdiagnosis of delirium and an inclusion of frail populations. The difference in dementia prevalence among the groups might also have influenced the outcome, since it is a risk factor and affects delirium prognosis.

Non-pharmacological interventions for delirium were generally applied together, encompassing several domains, and seem less effective in controlling symptoms. It was difficult to discriminate the effectiveness of each individual therapeutic approach.

Pharmacological intervention studies have generally included cholinesterase inhibitors, typical (haloperidol and droperidol) and atypical antipsychotics (olanzapine and others). Despite the fact that there have been few published studies, involving a small number of cases, pharmacological treatment significantly improved delirium duration and severity.

Rivastigmine, a cholinesterase inhibitor used for treatment of dementia, decreased the duration of delirium, improved cognitive function and reduced caregiver stress in older patients with ischemic stroke.^25^ Both olanzapine and haloperidol decreased the severity of delirium in elderly patients admitted to medical wards, and recovery was slightly faster with olanzapine.^24^ The use of droperidol reduced the length of hospital stay and increased postoperative delirium remission rate in elderly patients with colorectal cancer.^28^

Once old patients have developed acute cognitive decline, delirium must be diagnosed and promptly treated. The therapy includes detecting and treating underlying critical conditions, providing pharmacological control of agitation preferentially with atypical antipsychotics, improving cognitive function with cholinesterase inhibitors, and associating multiple interventions to hydrate, restore the sleep–wake cycle, provide early mobility, support visual and hearing deficits, provide temporal orientation and familiarity sensation, and improve communication. Therapeutic action in multiple domains can decrease severity of mental illness, time to delirium recovery, and guarantee better long-term cognitive function.
